# A qualitative study of bereaved relatives’ end of life experiences during the COVID-19 pandemic

**DOI:** 10.1177/02692163211004210

**Published:** 2021-03-30

**Authors:** Jeffrey R Hanna, Elizabeth Rapa, Louise J Dalton, Rosemary Hughes, Tamsin McGlinchey, Kate M Bennett, Warren J Donnellan, Stephen R Mason, Catriona R Mayland

**Affiliations:** 1 Department of Psychiatry, University of Oxford, Warneford Hospital, Oxford, UK; 2 Palliative Care Institute Liverpool, North West Cancer Research Centre, University of Liverpool, Liverpool, UK; 3 Department of Psychology, University of Liverpool, Liverpool, UK; 4 Department of Oncology and Metabolism, University of Sheffield, Sheffield, UK

**Keywords:** End of life, COVID-19, experience, support, dying, relatives, health professionals, palliative care, qualitative study

## Abstract

**Background:**

Meeting the needs of relatives when a family member is dying can help facilitate better psychological adjustment in their grief. However, end of life experiences for families are likely to have been deleteriously impacted by the COVID-19 crisis. Understanding how families’ needs can be met during a global pandemic will have current/future relevance for clinical practice and policy.

**Aim:**

To explore relatives’ experiences and needs when a family member was dying during the COVID-19 pandemic.

**Design:**

Interpretative qualitative study using semi-structured interviews. Data were analysed thematically.

**Setting/participants:**

A total of 19 relatives whose family member died during the COVID-19 pandemic in the United Kingdom.

**Results:**

In the absence of direct physical contact, it was important for families to have a clear understanding of their family member’s condition and declining health, stay connected with them in the final weeks/days of life and have the opportunity for a final contact before they died. Health and social care professionals were instrumental to providing these aspects of care, but faced practical challenges in achieving these. Results are presented within three themes: (1) entering into the final weeks and days of life during a pandemic, (2) navigating the final weeks of life during a pandemic and (3) the importance of ‘saying goodbye’ in a pandemic.

**Conclusions:**

Health and social care professionals can have an important role in mitigating the absence of relatives’ visits at end of life during a pandemic. Strategies include prioritising virtual connectedness and creating alternative opportunities for relatives to ‘say goodbye’.

**What is already known about the topic?**

- The final weeks and days of life is a stressful period for the whole family.
- Families cope and adjust better in bereavement when they are involved in the end of life experience of a dying family member, and are provided with detailed information from health and social care professionals about their relative’s condition.

**What this paper adds?**

- As a result of restricted visiting to hospital and care home settings during the pandemic, relatives relied on connecting virtually with their family in their final weeks of life, which could only happen when they were facilitated by health and social care professionals.
- Due to visiting restrictions imposed during the pandemic, relatives reported increased communication needs, such as more holistic information about their dying family member’s wellbeing, and psychological support.
- Relatives highlighted their need for practical and emotional support when a family member was at end of life, however for some families this need often outweighed observing the lockdown restrictions that were in place as a result of COVID-19.

**Implications for practice, theory or policy**

- Health and social care professionals were perceived to be instrumental to ensuring connectedness between patients and relatives at end of life during a pandemic.
- Proactive measures such as facilitating video and telephone calls between family members and providing detailed information reflecting holistic aspects of care should be prioritised.
- Despite the challenges and unpredictability of the pandemic, opportunities to ‘say goodbye’ to a family member who is dying is even more important for families, and should be facilitated if and where possible.

## Introduction

COVID-19 is likely to have a major impact on the experience of death and dying for families bereaved during the pandemic in the United Kingdom. To help avoid the spread of the virus and protect public health, access to general hospital settings, specialised palliative care units and residential care homes has been limited, including restrictions to the number of family members permitted to visit.^1^ Social, emotional and practical support has been constrained by the prohibition of individuals from different households mixing and controls on travel outside localities. Health and social care professionals are also heavily burdened by the current situation with pressures related to high numbers of seriously ill patients, equipment shortages, and moral distress regarding provision of appropriate person-centred care.^2^

When the needs of relatives are met at the time a family member is dying, they are reported to cope and adjust better in bereavement with improved psychological outcomes and satisfaction with end of life care.^3^ This includes clear and honest communication from health and social care professionals regarding a family member’s illness and declining health^4^; involvement in decision-making and other aspects of patient care^5^; and the opportunity for final contact with their family member before they die.^6^ Thus, the practical implications of the pandemic may have consequences for relatives’ physical and mental health, as well as their general wellbeing after their family member has died.

Exploration of relatives’ experiences will provide valuable insight as to how families managed and adapted when a family member was dying during the COVID-19 crisis. This will aid our understanding about how the needs of families can be facilitated during a pandemic and have direct ongoing relevance for clinical practice and policy. Additionally, lessons learned may have bearing on other situations where direct visiting may not be possible at end of life such as relatives being abroad or other infectious disease outbreaks.

### Aims and objectives

The current study aims to explore the experiences and needs of relatives when their family member was dying during the COVID-19 pandemic in the United Kingdom. The objectives of this study are to investigate:

- How relatives managed the final weeks of life when a family member was dying during the COVID-19 pandemic.
- Communication between relatives, their family member and health and social care professionals when a family member was dying during the COVID-19 pandemic.
- Relatives’ perceptions of how best they could have been supported when a family member was dying during the COVID-19 pandemic.

## Methods

An interpretative qualitative design using semi-structured interviews.^7^ This study is reported following the Consolidated Criteria for Reporting Qualitative Research (COREQ) guidelines.^8^

### Context

This study was embedded within a national UK survey of bereaved relatives’ views about end of life care experiences. The wider national study is part of an international collaborative project, involving 20 countries, and led by Erasmus University, Rotterdam.

### Participants

A total of 19 relatives participated in the study between July and December 2020. Convenience sampling identified participants from a database of individuals who expressed an interest to be interviewed after completing a COVID-19 questionnaire within our wider study. Individuals were considered eligible if they had experienced the death of a family member during the COVID-19 pandemic, were aged 18+, and resided in the United Kingdom. Purposive sampling techniques were used in later interviews to include a wide range of family members (in terms of age, gender and relationship to the deceased). Eligible participants were contacted by 1 of the researchers [RH] via email, of which 28 potential participants did not respond to the invitation and 1 declined. It is unclear why eligible participants did not respond to the invitation, as from an ethical perspective no follow-up took place following the invitation. Interested and willing relatives confirmed participation [with RH]. Written consent was obtained prior to the interview.

### Data collection

Semi-structured interviews were carried out. This method was considered most appropriate for providing rich data surrounding an individual’s experience. A topic guide was developed, informed by the research aims and objectives and the research team who have a wealth of combined palliative care and bereavement research and clinical experiences. The topic guide was iteratively modified as necessary to ensure follow-up with categories in subsequent interviews (Table 1). Interviews were completed by two authors [RH, TM], neither of whom had prior relationships with the participants. To demonstrate reflexivity, both researchers [RH, TM] recorded reflections after each interview. These were discussed at regular team meetings with all authors throughout the data collection period. Interviews were conducted on Zoom (*n* = 4) or telephone (*n* = 15), audio-recorded and lasted between 20 and 98 min (*mAvg* = 56.3 min). Interviews were completed when no further categories were identified.

**Table 1.** Semi-structured topic guide used to guide the conduct of the study.

**Initial topics based on the study aims and objectives**
Exploration of how relatives managed the final weeks and days of life
Perceptions of how the pandemic impacted the final weeks and days of life
Communication between the relative, their dying family member and healthcare team in the final weeks and days of life
Perceptions of how best the relative/family could have been supported at end of life
**Sample of additional topics as categories were identified**
Staying connected with the dying family member in the final weeks and days of life
Virtual technology and connecting with the dying family member
The role of support networks
Saying goodbye to the dying family member during a pandemic
Family-centred care

### Data analysis

Audio-recordings were transcribed verbatim and managed using NVivo V.12. Transcripts were not returned to the participants but were verified by one researcher [RH]. Braun and Clarke’s^9^ thematic analysis framework was used to analyse the data. This framework was considered most appropriate as it is a flexible method useful to exploring individual experiences, perspectives and opinions. Initially, the first author [JRH] read and reread the transcripts to gain a sense of each relative’s story. To ensure rigour, credibility and trustworthiness, three authors [ER, LD, RH] also read the transcripts. Following line by line scrutiny of the transcripts, the first author [JRH] manually coded the data, detailing inductive descriptive codes by marking similar phrases or words from the relatives’ narratives. The first author [JRH] collated the codes and identified where some of them merged into themes using mind maps. Due to overlap of data in some themes, all codes and themes were independently analysed by two co-authors [ER, LD], which resulted in the removal of one broad theme and inclusion of one sub-theme. Themes were discussed and refined through critical dialogue with all authors.

### Ethical considerations

Participants were provided with oral and written information about the study and provided oral and written consent. In view of the potentially emotive subject, participants were aware of their right to withdraw from the study, as well as the option to pause, terminate or reschedule the interview. Correspondence between the researcher [RH] and participants took place via University email, and all emails containing personal information were deleted at the earliest convenience. Participants were provided with information of support organisations as part of the study’s debrief. Data protection procedures were observed and assurances of confidentially were provided. Ethical approvals were obtained from University of Liverpool Central University Research Ethics Committee [Ref: 7761].

## Results

A total of 19 relatives were recruited, 12 who were female and 7 were male. The relative’s relationship with their family member varied, to include spouse/partner (*n* = 4), adult child (*n* = 11), son/daughter in-law (*n* = 2), niece (*n* = 1) and grandchild (*n* = 1) of the deceased. Interviews took place between 2 and 6 months after the death. Sample characteristics are reported in Table 2.

**Table 2.** Characteristics of the 19 participants included in the study.

Variable	*N*
**Gender**	
Female	12
Male	7
**Relationship to the family member**	
Spouse/partner	4
Adult child	11
Adult grandchild	1
Son/daughter in-law	2
Niece	1
**Ethnicity of relative/deceased**	
White (English/Welsh/Scottish/Northern Irish/British)	19
**Location of relative/death**	
England	14
Scotland	4
Wales	1
Northern Ireland	0
**Place of death**	
Hospital	10
General ward (*n* = 3)	
Intensive care unit (*n* = 4)	
Coronavirus ward (*n* = 3)	
Care home	9
**Chronic condition of deceased family member**	
Dementia	8
Cancer	4
Heart failure	3
COPD	2
Renal disease	1
None identified	1
**Age of relative**	
20–29	1
30–39	2
40–49	1
50–59	8
60–69	6
70–79	1
**Age of dying family member**	
50–59	1
60–69	3
70–79	3
80–89	9
90+	3

Most reported their family member had been living with a chronic illness (including dementia, cancer, heart failure, COPD and renal disease) and coping with the ‘ups and downs’ of treatment and side-effects at home or a care home for a period of months or years. At a point during the pandemic, the deceased had rapid signs of disease progression and deterioration; the majority also tested positive for COVID-19 (*n* = 13).

The data below is representative of relatives’ experience of the final weeks and days of life, of which the dying family member was receiving care at the care home (*n* = 9) or hospital [general ward (*n* = 3), intensive care unit (*n* = 4), coronavirus ward (*n* = 3)]. Overall, relatives described both positive and negative perceptions of care delivery. Where negative aspects were described, some relatives framed their responses in the context that the health and social care professionals were ‘doing their best given the situation’, and reflected on areas they perceived could have been better. Overall, three themes were identified: (1) entering into the final weeks and days of life during a pandemic, (2) navigating the final weeks of life during a pandemic and (3) the importance of ‘saying goodbye’ in a pandemic.

### Theme 1: Entering into the final weeks and days of life during a pandemic

Relatives reported feeling numb when they first learned of the news that their family member was displaying symptoms of COVID-19, with concerns about how the (likely) virus might impact on the family member’s underlying condition. While some family members were showing few signs of physical deterioration as a result of their underlying condition, many were admitted to hospital to help with management of their symptoms, which may have been COVID-19 related. Relatives reported this as a distressing period with a conflict between their desire to be with their family member and the COVID-19 restrictions which meant they were unable to do so. Most relatives reported they received a telephone call from the hospital or care home to inform them that their family member’s condition had rapidly deteriorated and was expected to die within the next few weeks or days.

*“This was the first time I wasn’t able to go in the ambulance with him. I had a bad feeling after he tested positive. His breathing wasn’t good at all. I started to worry that this was the beginning of the end” [Relative 01; spouse of the deceased; hospital death]*

### Theme 2: Navigating the final weeks of life during a pandemic

The time-period between receiving the news that their family member was going to die soon and the death occurring varied for families, but the experience of uncertainty was universal. A number of factors appeared helpful for relatives when their family member was in the final weeks of life during the COVID-19 pandemic. These are discussed further under three sub-themes: (1) staying connected with the family member, (2) the availability of support networks and (3) clear communication and support from health and social care professionals.

#### Sub theme 1: Staying connected with the family member

Although a few relatives were initially able to stay connected with their family member via a personal mobile phone, most described their family member as ‘too poorly’ and less able to communicate with them. Nonetheless, it was important for relatives to be part of their family member’s final weeks and days of life, to which the extent and manner of this involvement varied between families. Most families were unable to visit their family member who was dying until their final hours, although some reported being able to spend time with them in the final weeks of life at either the hospital or care home. This enabled relatives to help with personal aspects of care such as combing their family member’s hair or simply being able to hold their hand as they slept; relatives reflected on the importance of being able to provide this care to their family member. For others, physical closeness or contact was not possible, leaving relatives only able to see their dying family member from the window of their care home, for example. While some relatives were grateful for this opportunity, others reported this was very upsetting as they were unable to be physically close or touch.

*“I was up against the window as close as I could get. He was talking to me which I could hear but he couldn’t hear me back, so it became distressing for both of us” [Relative 15, adult child of deceased, care home death]*

Health and social care professionals often offered FaceTime or WhatsApp video calls between relatives and with their family member at the care home or hospital in the final weeks of life. Relatives reported these video calls were very important as it enabled the family member to continue to be involved in aspects of everyday life within the family.

*“He [family member] was able to see his wee grandson and being quite playful and laughing and joking on a camera phone, which was lovely as it was our only way of seeing him” [Relative 16, adult child of deceased, hospital death]*

However, virtual communication often presented challenges for some family members and their relatives, such as difficulties hearing or with WiFi connection. Other relatives reported wanting calls with their family member in the hospital or care home, but these were not facilitated. These relatives perceived that the healthcare setting or care home did not have devices available to facilitate these calls, or health and social care professionals did not have the time to offer these aspects of care.

*“I don’t think they [care home] had iPads or tablets at that time. They never offered it and we didn’t know to ask either. I’m not sure they would have had the time for that” [Relative 04, adult child of deceased, care home death]*

#### Sub theme 2: The availability of support networks

Relatives reported a need for additional assistance for themselves and other family members to help manage day to day tasks when their family member was in the final weeks of life. It was valued when other relatives or neighbours helped with practical tasks such as shopping or collecting medications, particularly those who had other caring responsibilities or had reduced ability to go out themselves. While some relatives, particularly those that lived alone, highlighted a desire for close family members to be with them to provide comfort in those final weeks, others reported some close relatives moved in to provide such support. The need for comfort outweighed the lockdown restrictions that were in place as a result of the pandemic. However, it appeared most relatives had a lack of available support networks and struggled by themselves as a result of shielding, lockdown restrictions or other relatives living too far away.

*“It was obvious we were breaking the rules. If we hadn’t broken the rules, I don’t think my dad [a well parent] would be here. I really don’t. He couldn’t have coped. [Relative 07, adult child of the deceased, hospital death]*

#### Sub theme 3: Clear communication and support from health and social care professionals

As most relatives were unable to visit the care home or hospital to see their family member in their final weeks of life, they relied on telephone updates from health and social care professionals regarding their condition. While some relatives reported receiving a daily phone call from the care home or hospital regarding their family member’s health, most reported they had to seek out this information themselves, which presented them with challenges. This included laborious efforts associated with getting in contact with the hospital ward or the appropriate health and social care professional(s) caring for their family member, which was highlighted as distressing for relatives at an already highly stressful time.

*“I was told I would get an update in two hours, and I think three hours passed and still nothing. I just wanted to know how he was doing. Just not knowing was stressful in itself. So, I decided to call, but then I spent so long on the phone and was being passed from pillar to post as they couldn’t find somebody who was caring for him to tell me anything” [Relative 18, daughter-in-law of deceased, hospital death]*

The level of information provided to relatives varied. Most relatives reported wanting information regarding the decline in their family member’s health, estimated timelines of when the death may happen, information pertaining to their symptoms and how these were being managed, as well as updates to aspects of personal care, such as if they had been given a shave or if they had been eating or drinking. However, often relatives were only informed their family member was ‘comfortable’, with few opportunities provided for them to ask questions to the health and social care team. Some relatives reflected they would have liked guidance and support from health and social care professionals in relation to telling children (<18 years old) that their parent, grandparent or relative was going to die soon. However, health and social care professionals rarely asked about the patient’s family and social network, so children affected by the family member’s illness and imminent death were not identified by professionals nor was advice provided from them to support families with these conversations. The main content of communication between health and social care professionals and families predominately focused on the family member’s physical care, rather than the emotional implications for their wider network.

*“I had a million and one questions about how Mum was doing but I didn’t get a chance to ask them. I was no sooner on the phone than I was off it again. Then the rest of the family were looking at me for information and I didn’t have anything to tell them. We couldn’t see Mum ourselves, so we just felt quite in the dark at that time” [Relative 02, adult child of deceased, care home death]*

Some relatives reported it was challenging to understand some of the information shared with them on the telephone from the hospital or care home, as they felt the personal protective equipment (PPE) worn by health and social care professionals impacted their ability to clearly hear what was being communicated, which was highlighted as stressful. Some relatives found it helpful when health and social care professionals included other family members in communication about care, as this helped to ensure consistency of information and understanding, but it seemed this rarely happened.

*“I was speaking to the nurse who was looking after him who obviously was wearing PPE to protect themselves, but it was really distressing because I couldn’t properly hear her and wasn’t really sure of the outcome of the conversation” [Relative 08, spouse of deceased, hospital death]*

### Theme 3: The importance of ‘saying goodbye’ in a pandemic

It was important for relatives to have the opportunity to physically be with their family member before they died. Most had not seen their family member for a period of weeks or months due to the pandemic and desired an opportunity to ‘say goodbye’ before it was ‘too late’. Relatives highlighted this was their ‘last chance’ to see their family member as they were unable to have an open coffin in the funeral parlour or at home during the immediate bereavement period.

*“The actual fact that we could go there and see her, because we hadn’t seen her for so long. It was important being able to touch her one last time and talk to her” [Relative 11, adult child of deceased, care home death]*

Spending time with their family member in the final days and hours of life varied for families. While some relatives reported being able to spend time with their family member in the hospital or care home at the end, others had to make a difficult decision not to visit. Factors contributing towards this decision included fears of ‘catching the virus’ in the hospital or care home and perceptions that wearing PPE would impact the quality of their interaction with the family member. The requirement to self-isolate for 10–14 days after a visit to see a family member prohibited some relatives who had caring responsibilities or other commitments from visiting their family member.

Relatives who were unable to be with their family member when they were recognised to be actively dying reported it helpful when the hospital or care home facilitated a telephone call between them and their loved one, often with a health or social care professional holding the phone to the family member’s ear as the relative ‘said goodbye’. It appeared this call only took place when relatives asked for them, but rarely happened and was sometimes discouraged by health and social care professionals because the dying family member ‘would not respond’, or perceptions it would be too distressing for the dying family member who had cognitive decline. Families found it comforting to know that someone was with their family member as they were dying, such as a health or social care professional or chaplain within the hospital or care home.

*“It was four days before he died, and I said to Mum ‘why don’t you ask the nurses if they can put the phone to his ear and you can say the things you want to’. So, she did and the nurse on the telephone basically said, ‘there’s no point in doing that [I’m] sure he won’t be able to hear you’, which we both were quite upset by” [Relative 03, adult child of deceased, hospital death]*

It was reported in the days leading up to the death relatives often expressed to the hospital or care home their desire to be with the dying family member, but were frequently informed by healthcare professionals ‘we’re not at the end yet’, ‘it’s not the time yet to come in and say goodbye’ or ‘we still have time’. Despite these requests, most participants reported by the time they were informed their family member was going to die soon, they had ‘not made it to the hospital or care home in time’ and were not able to say goodbye to their family member.

*“We got the call saying, ‘how quick can you get over because your dad, we don’t think he’s going to last long’. So, I was halfway on the road, as it’s a 10-mile journey and got the call telling me he died” [Relative 17, adult child of deceased, care home death]*

## Discussion

Findings highlighted relatives’ desire and need to maintain a connection to their family member when they were in the final weeks and days of life. Similar findings have been reported in the literature.^10–12^ While many families were unable to physically spend time with their dying family member as a result of the pandemic, it was possible to achieve connectedness through video or telephone calls. However, these necessitated facilitation by health and social care professionals and it appeared from this study that these virtual interactions were rare; this may reflect a lack of devices or available signal in the hospital or care home.^13^ Other explanations may reflect the reliance on health and social care professionals who may have lacked adequate time to provide these aspects of supportive care,^14^ or underestimated the importance of this communication for relatives when a family member is at end of life.^15,16^ Being provided with the opportunity to connect with a dying family member in the final weeks and days of life may facilitate a better adjustment for relatives in their bereavement.^17,18^ There is a need for relatives to have contact with their family member in the final weeks of life, particularly when there is a reduced availability of visiting. In this pandemic, the situation was compounded by the pressure on health and social care professionals to provide these aspects of care, and the absence of end of life care volunteers who would otherwise have an instrumental role in enabling this connectedness.^19^

Relatives reported the importance of support networks available to provide emotional support and help with the practical aspects of daily tasks when a family member was in the final weeks and days of life. Similar findings have been reported in the literature.^20,21^ While this may be inhibited or complicated by COVID-19 restrictions, health and social care professionals could encourage relatives to engage with alternative, virtual support networks in the final weeks and days of life, to provide such emotional and practical support.

It was important for relatives to be provided with clear and detailed information from health and social care professionals regarding their family member’s condition throughout the final weeks and days of life. This included information regarding their health decline,^4,22^ symptom management,^23,24^ and personal aspects of care.^23^ It seemed there was a disparity between relatives’ desired and required level of information from health and social care professionals about their family member’s care and what was provided in practice. It is possible health and social care professionals underestimated how much information families wanted regarding their dying family member, and highlights the importance for professionals to create opportunities to ask questions. It may be that the health and social care team were unprepared or lacked the requisite communication skills to address these often complex and emotionally laden conversations, particularly when not face-to-face.^4^ Further, the emotional labour of caring for increased numbers of patients at the end of life during the COVID-19 crisis is likely to have taken its toll on health and social care professionals.^25^ Future research could explore how communication between health and social care professionals and relatives when a family member was at end of life varied between different health and care settings, to better understand best practices surrounding communication at end of life during a pandemic.^26,27^

Some relatives reflected that they would have welcomed guidance from health and social care professionals surrounding how to prepare children for the death of a relative. It appeared from the findings that such family-centred care was lacking as communication from healthcare teams was predominately focused on clinically-driven care. This may reflect health and social care professionals’ perception that providing families with advice and guidance in relation to dependent children when a relative is at end of life does not constitute part of their role, or that another professional was providing it.^28^ Children cope and adapt better when they are prepared for death.^29,30^ It is important that health and social care professionals engage in family-centred conversations with relatives at end of life.^28,31,32^

Literature highlights that relatives’ desire the opportunity for time with their family member in the final weeks and days of life.^6,10^ Alongside this, other studies have reported that providing personal aspects of care or holding a family member’s hand has provided comfort and enabled connectedness between relatives and a dying family member at end of life.^33–35^ The findings of this study highlighted it was especially important for relatives to spend some time with their family member at the end of life as most had not seen them for a period of weeks or months due to the pandemic, and they would not have the opportunity to view the body in the immediate bereavement period. Most relatives did not have the chance for this time with their family member before they died as the findings suggest death approached rapidly at the end. It is possible that due to the unpredictability of COVID-19, health and social care professionals did not expect death would be so imminent and there would be ‘more time’.^36,37^ Alongside this, the government restrictions of visiting at end of life in institutional settings as a result of COVID-19 may have presented health and social care professionals with a dilemma of balancing the importance of relatives wanting to be with their dying family member and the unpredictability as to when is the time to enable this to happen. Families cope better long-term when they have an opportunity to say goodbye to their dying family member^18,38^; this indicates that health and social care professionals should consider offering a family visit at an earlier opportunity, rather than waiting until death is expected within hours or days. The absence of this final contact for many relatives can lead to poorer outcomes for families,^39^ and therefore has implications for post-bereavement services for relatives.

### Strengths and limitations of the study

This is a timely study that reports recommendations for health and social care professionals as they provide end of life care during a pandemic from the perspective of bereaved relatives. Findings are limited to relatives whose family member died in a care home or hospital and do not represent the experience and needs of families when a family member died in other settings such as home or hospice, even though attempts were made to include these relatives. While this study did not identify differences between those relatives whose family member died in the hospital or care home, future research could explore this phenomenon further. Also, most of deceased had an underlying chronic condition and the findings do not account for the experience of families when someone previously well died of COVID-19. Alongside this, most of the deceased would be considered within a frail elderly category.^40^ This study is limited to the relatives’ experiences whose family member died during the COVID-19 pandemic in United Kingdom. There did not appear to be any challenges in relation to conducting sensitive interviews with bereaved relatives using virtual platforms, and the researchers did not find any differences between conducting the interview on Zoom or telephone. Findings are limited to an ethnically homogenous white British population. Future research could focus on the experiences of end of life for families from Black, Asian and Minority Ethnic communities.

## Conclusion

Through the lens of bereaved relatives, this research has provided insight into the experience of families when a family member was dying during the COVID-19 pandemic. In the absence of physical contact, health and social care professionals can have an important role to ensuring connectedness between patients and their families. This includes facilitating video and audio calls between relatives and their dying family member and providing the family with detailed updates regarding their dying family member’s physical condition. Also, it is pertinent for health and social care professionals to provide psychosocial and other aspects of care such as whether the family member has been eating or drinking, or other information important to the relative. Especially during periods of social distancing and lockdown restrictions, there is a need for health and social care professionals to create opportunities for relatives to ‘say goodbye’ to their family member at end of life before it is ‘too late’.
